# MR1- and HLA-E-Dependent Antigen Presentation of *Mycobacterium tuberculosis*

**DOI:** 10.3390/ijms232214412

**Published:** 2022-11-19

**Authors:** Se-Jin Kim, Elham Karamooz

**Affiliations:** 1 Department of Pulmonary and Critical Care Medicine, Oregon Health & Science University, Portland, OR 97239, USA; 2 Department of Molecular Microbiology and Immunology, Oregon Health & Science University, Portland, OR 97239, USA; 3 Medical Scientist Training Program, Oregon Health & Science University, Portland, OR 97239, USA

**Keywords:** mycobacteria, immunology, antigen presentation

## Abstract

MR1 and HLA-E are highly conserved nonclassical antigen-presenting molecules. They can present antigens derived from *Mycobacterium tuberculosis* to a distinct subset of MR1-restricted or HLA-restricted CD8+ T cells. MR1 presents small microbial metabolites, and HLA-E presents peptides and glycopeptides. In this review, we will discuss the current understanding of MR1 and HLA-E antigen presentation in the context of *Mycobacterium tuberculosis* infection.

## 1. Introduction

Before the COVID-19 pandemic, *Mycobacterium tuberculosis* (Mtb) was the leading cause of infectious disease mortality in the world [1]. The only available vaccine for Mtb is Bacille Calmette–Guérin (BCG), which provides some protection against Mtb infection in children, but this protection does not continue in adulthood, possibly due to the lack of a booster vaccine [2]. Therefore, there is a great deal of interest in understanding the immune response to Mtb in order to develop Mtb vaccines that are more effective than BCG. Most people infected with Mtb are asymptomatic, and control of the infection is largely due to the cellular immune system.

The cellular immune system includes macrophages, CD4+ T cells, and CD8+ T cells, and each of these cell types plays a role in controlling Mtb. Traditional CD8+ T cells are restricted by MHC-Ia (HLA-A, -B, and -C) and are able to sample the intracellular environment for self- and non-self-peptides [3,4]. These genes are highly polymorphic across the population, with thousands of alleles identified [3]. The structure of MHC-Ia is composed of 3 alpha domains, where the alpha 1 and alpha 2 domains form the antigen-binding pocket for a peptide of 8-10 amino acids [3,4] and the alpha 3 domain binds β-2-microglobulin (β_2_m) [4]. MHC-Ia molecules remain in the endoplasmic reticulum (ER) and are stabilized by important chaperone proteins, collectively referred to as the peptide-loading complex (PLC) [3,4]. Peptides can enter the ER via the transporter associated with antigen processing (TAP), a key component of the PLC, and once an appropriate peptide binds MHC-Ia, the structure is stabilized and translocates to the cell surface [3,4].

Multiple researchers have demonstrated the importance of CD8+ T cells in Mtb immunity. First, in a landmark paper, mice lacking β_2_m had a significant increase in mortality compared with controls [5]. This finding was corroborated in a study of TAP1 knockout mice, which were deficient in classical CD8+ T cells [6]. In addition to having direct cytotoxic effects, CD8+ T cells are producers of pro-inflammatory cytokines, such as IFN-γ and TNF-α. In mice, the disruption of either IFN-γ or TNF-α caused a significant increase in mortality after Mtb infection [7,8,9]. These two proinflammatory cytokines also play a critical role in humans. For example, the TNF-α inhibitor infliximab was associated with the reactivation of latent Mtb, and mutations of the IFN-γ receptors were associated with a variety of mycobacterial infections [10,11].

In addition to MHC-Ia, there are several conserved, nonclassical, antigen-presenting molecules that are capable of presenting different types of Mtb antigens to different T cell subsets. These molecules are MHC class I-related (MR1), HLA class I histocompatibility antigen, alpha chain E (HLA-E), CD1, and butyrophilin 3A1 [12]. In a study using different knockout mice (β_2_m, TAP, CD8, and perforin), Sousa et al. showed that CD8+ T cells were essential for protection against Mtb and that there was a distinct contribution from nonclassical CD8+ T cells [13]. In subjects with latent Mtb, the majority of CD8+ T cells identified via a limiting dilution assay were nonclassical T cells [14].

In this brief review, we will focus on MR1 and HLA-E antigen presentation as CD1 and butyrophilin 3A1 have been previously reviewed [15,16]. Both MR1 and HLA-E are structurally similar to MHC-Ia, although MR1 is located on chromosome 1 while HLA-E is located on chromosome 6 with the other MHC-Ia alleles, and both molecules are found on a variety of nucleated cells [17,18,19]. Despite structural similarities, the antigens presented by these two molecules differ dramatically. Here, we will highlight what is known about MR1 and HLA-E antigens and antigen presentation in the setting of mycobacterial infection.

## 2. MR1 and MR1-Restricted T Cells

MR1 was first discovered in 1995 and found to be similar to MHC-Ia, but its function was unknown [17]. Subsequent studies revealed that MR1 can bind β_2_m and the PLC [20,21]. In 2003, Treiner et al. identified that a subset of T cells expressing a TRAV1-2 T cell receptor (TCR) α chain were restricted by MR1 [22]. These T cells were highly prevalent at mucosal sites and were named mucosal-associated invariant T (MAIT) cells [22]. Despite the identification of these T cells, it was not until 2010 that Gold et al. and Le Bourhis et al. determined that MAIT cells have the ability to detect microbial infections, including Mtb, other mycobacteria, yeast, and a variety of Gram-positive and Gram-negative bacteria [23,24]. In response to these infections, MAIT cells produce IFN-γ and TNF-α [23,24].

## 3. MR1 Ligands and Antigens

The highly conserved sequence of MR1 across the population suggested that MR1 antigens are conserved. The fact that TAP was not required for the selection of MAIT cells indicated that the antigens were not peptides [22,23,24,25]. However, there was a significant challenge in identifying MR1 antigens, and the identification of these antigens remained elusive. In a seminal study, Kjer-Nielsen et al. demonstrated that MR1 can bind vitamin B metabolites [26]. This was discovered when the investigators found that MR1 formed stable complexes with β_2_m in the cell culture medium RPMI-1640 [26]. Using mass spectrometry, the ligand was identified as a photodegradative product of folic acid (vitamin B9), specifically the metabolite 6-formyl pterin (6-FP) [26]. Even though 6-FP was bound to MR1, Jurkat cells transduced with a MAIT TCR (Jurkat.MAIT) were not activated. Instead, 6-FP assisted in the stabilization of MR1 on the cell surface [26]. These data indicated that although 6-FP can bind MR1, it cannot activate MAIT cells; therefore, other activating ligands must be present.

To identify MR1 ligands that activate Jurkat.MAIT cells, Kjer-Nielsen et al. conducted mass spectrometry on MR1 refolded with *Salmonella enterica* serovar *typhimurium* supernatant and determined that ribityllumazines, which are intermediates in the riboflavin biosynthesis pathway, were the antigens MR1 was presenting [26]. Since mammals do not make riboflavin, these antigens are inherently non-self. Subsequent work identified additional biochemical antigens that are even more potent than the ribityllumazines. Corbett et al. found that 5-amino-6-D-ribitylaminouracil (5-A-RU), an intermediate of the riboflavin biosynthesis pathway, combined with glyoxal or methylglyoxal, both byproducts of glycolysis, form 5-(2-oxoethylideneamino)-6-D-ribitylaminouracil (5-OE-RU) or 5(2-oxopropylideneamino)-6-D-ribitylaminouracil (5-OP-RU), respectively [27]. These are the most potent MR1 antigens. Functionally, 5-OP-RU is critical for MAIT cell development based on data showing that 5-OP-RU produced in the gut by commensal bacteria could reach the thymus and was necessary for MAIT cell development [28]. With respect to inhibitory ligands, acetyl-6-FP has been identified as a more potent inhibitory molecule than 6-FP, and it is widely used in assays that measure MR1 surface stabilization [29].

Despite the potency of 5-OP-RU, other studies suggest that MR1 is capable of binding to a diverse set of ligands. First, in MR1-restricted T cells, differences in TCR sequences led to varying MR1-antigen complex recognition [30,31]. Next, an MR1-restricted T cell clone was characterized that detected *Streptococcus pyogenes*, a microbe that lacks the riboflavin biosynthesis pathway [32]. The antigen presented by MR1 from *Streptococcus pyogenes* is still unknown. Using mass spectrometry, Harriff et al. identified a unique antigen from *Mycobacterium smegmatis* called photolumazine I [33]. This antigen was not identified in *Escherichia coli*, indicating that there are distinct ligands captured by MR1 from different microbes. Moreover, when photolumazine I was tested against different MAIT cell clones, only certain TCRs were capable of recognizing the MR1-photolumazine I complex [33]. In addition to diversity in microbial antigens, Keller at al. found that several small molecules, including the pharmaceutical drug diclofenac, can activate Jurkat.MAIT cells [34]. Taken together, these data show that there is more diversity in the range of MR1 ligands than initially thought.

## 4. MR1 Antigen Presentation of *Mycobacteria*

The presentation of phagocytosed antigens on MHC-Ia is called cross-presentation [35]. While MHC-Ia antigen loading is classically defined as occurring in the ER with the PLC, cross-presentation happens outside of the ER through multiple potential mechanisms [35]. Unlike MHC-Ia, there is very little MR1 expressed on the cell surface, suggesting regulatory mechanisms at play to prevent unloaded MR1 from reaching the plasma membrane [21]. Similar to MHC-Ia, MR1 can be loaded with an antigen within the ER and also outside of the ER [36]. The ER pathway was defined in C1R cells, where under basal conditions, MR1 is mostly retained in the ER, as shown biochemically using an endoglycosidase H assay [37]. Once an appropriate antigen is added, the antigen forms a Schiff base with lysine 43 of MR1 [26]. This bond neutralizes a positive charge and allows MR1 to leave the ER and reach the cell surface. When lysine 43 mutates to alanine, MR1 is measured on the cell surface at levels similar to wild type MR1 bound to a ligand [37]. When lysine 43 mutates to arginine, to retain the positive charge and prevent a Schiff base from forming, MR1 remains in the ER despite the presence of a ligand [37]. A separate study using a fluorescent MR1 ligand and a proximity ligation assay provided direct evidence that exogenously delivered antigen reached the ER [38]. Furthermore, confirming an earlier study of MR1 [21], investigators found that MR1 interacts with the PLC and coprecipitates with the chaperone TAPBPR and that the deletion of both TAPBPR and tapasin substantially reduced MR1 surface expression [38].

While it is clear that MR1 can be retained and loaded in the ER, it is less clear that this represents the sole antigen presentation pathway in the context of an intracellular Mtb infection. The airway epithelial cell line, BEAS-2B, is efficient at presenting Mtb to MAIT cells and the presentation of Mtb antigens by MR1 requires intracellular infection [39]. In BEAS-2B cells, overexpressed MR1 tagged with GFP localized to the ER and to late endosomal compartments positive for Rab7 and LAMP1 [40]. The addition of 6-FP caused a substantial increase in MR1 surface stabilization, consistent with the previous observation of 6-FP in C1R cells [29,40]. In order to characterize the relevance of endosomal trafficking, a lentiviral shRNA library of 114 trafficking molecules was screened by infecting BEAS-2B cells with Mtb and quantifying IFN-γ production from a MAIT cell clone [40]. This approach identified several key endosomal trafficking molecules, including Rab6 and VAMP4, both of which are implicated in Golgi trafficking, and also syntaxin 18, which functions in ER-to-Golgi transport [40]. Huber et al. expanded on the role of Rab6 and found that it was important for the transport of MR1 from the cell surface to the Golgi, indicating a potential mechanism for MR1 recycling [41].

While knockdown of syntaxin 18, VAMP4 and Rab6 affected Mtb antigen presentation, only syntaxin 18 knockdown affected MR1 surface stabilization by 6-FP, suggesting distinct antigen presentation pathways between exogenously delivered antigens and an intracellular infection [40]. In a subsequent paper, knockdown of the trafficking protein syntaxin 4 affected exogenous antigen presentation without having any effect on intracellular Mtb presentation to a MAIT cell clone [42]. In addition, while overnight pretreatment of BEAS-2B with 6-FP resulted in the enhanced MR1 presentation of exogenous ligands, this effect was not seen with Mtb infection [42], supporting the notion of different pathways for an intracellular infection.

## 5. Clinical Implications of MR1 and *Mycobacterial* Infections

Some of the earliest data showed that MAIT cells are decreased in the peripheral blood in Mtb infection [23,24]. One hypothesis for this finding is that these T cells are being recruited from the blood to the lung to fight infection [43]. However, in rhesus macaques, pulmonary Mtb infection resulted in a variable increase in MAIT cells in the lung [44]. In humans, the importance of MR1 in the control of Mtb was suggested in a study showing that an MR1 single-nucleotide polymorphism was associated with Mtb meningitis [45]. Recently, investigators identified an individual with a homozygous mutation to MR1 that cannot bind 5-OP-RU [46]. This mutation, R9H, caused the failure of MAIT cell development, and susceptibility to viruses and intestinal infections. Although the individual had no history of tuberculous or nontuberculous mycobacterial infections, there was an expansion of γδ T cell populations to compensate for the loss of MAIT cells [46]. It is not known whether this contributed to protection from infections.

To determine if the population of MAIT cells could be expanded, three different groups conducted studies using the MR1 antigen 5-OP-RU. In a mouse model, 5-OP-RU and a Toll-like receptor (TLR) agonist increased the number of MAIT cells in the lungs of BCG-infected animals [47]. There was decrease in BCG growth compared with controls but no such reduction when mice were infected with Mtb [47]. A separate study in mice found that treatment with 5-OP-RU and a TLR agonist increased MAIT cells in the lungs, but this expansion of T cells did not reduce the Mtb load on intranasally infected mice [48]. In a different study, 5-OP-RU treatment helped mice with chronic Mtb infection by expanding the number of MAIT cells, and there was decreased bacterial load in an IL-17A-dependent manner [49]. However, 5-OP-RU treatment before Mtb infection was of no benefit and in fact impaired conventional T cell responses. Finally, in a recent study of BCG vaccination, investigators observed no changes in the frequency of MAIT cells in adults re-vaccinated with BCG [50]. Altogether, it is clear that an MR1-based approach to mycobacterial therapeutics will require additional study to better understand how to harness these T cell responses.

## 6. HLA-E and HLA-E-Restricted T Cells

HLA-E is a nonclassical molecule that has limited polymorphism diversity with two major alleles, HLA-E*01:01 and HLA-E*01:03 [51]. These two alleles account for most of those in humans and differ at one amino acid site [51]. This variation is at position 107, where it is arginine for HLA-E*01:01 and glycine for HLA-E*01:03. This difference influences the binding affinity of the peptide and thermal stability of the complex [52]. The most well-known function of HLA-E is to interact with CD94/NKG2A receptor, which leads to the inhibition of NK cell-mediated lysis [53,54]. However, HLA-E can also present antigens from a variety of bacterial and viral infectious pathogens [55]. HLA-E-restricted T cells have varied effector profiles. In a patient with latent Mtb, a CD8+ HLA-E-restricted T cell clone demonstrated a Th1 cytokine profile with IFN-γ production in response to stimulus using Mtb-infected antigen-presenting cells [56]. However, IFN-γ expression was not universally observed when investigators expanded HLA-E-restricted T cells using Mtb peptides [57]. Other studies identified HLA-E-restricted T cells with Th2 and Treg phenotypes, which expressed IL-4, IL-5, IL-13, and IL-10 [57,58,59,60]. These findings indicate potential properties of HLA-E-restricted T cells to become Th1 or Th2-like cells and express regulatory cytokines to control pathogenic inflammation from Mtb infection. 

The first identified peptides for HLA-E were derived from MHC-Ia leader sequences, which includes the canonical VL9 sequence [61]. This binding of leader peptides to HLA-E inhibits NK cell-mediated lysis. A notable difference between HLA-E and MHC-Ia peptide binding is that the HLA-E binding pocket is less flexible, which initially suggested a conserved peptide repertoire [62]. Additionally, small changes in the peptide sequence can substantially change the affinity of HLA-E for CD94/NKG2 receptor even though the overall structure of the peptide and HLA-E complex is not dramatically different [63].

*Cytomegalovirus* (CMV), *Salmonella*, and Mtb can induce HLA-E-restricted T cell responses [55]. A number of different Mtb peptides that bind HLA-E have been identified [57,64,65,66]. Using mass spectrometry, McMurtrey et al. identified 28 Mtb-derived peptides, many of which were derived from the Esx family of proteins [64]. Unlike leader peptides, the Mtb-derived peptides had a length of 8-20 amino acids. When these peptides were used to test T cell responses from 16 different donors, 12 of the peptides elicited HLA-E-restricted T cell responses as determined by IFN-γ ELISPOT assay [64]. Harriff et al. identified a specific glycopeptide from Mtb that was detected by an HLA-E-restricted T cell clone [65]. This antigen was derived from MPT32 and required Mtb-specific O-linked mannosylation for recognition. Lastly, a recent study demonstrated that a high-throughput binding assay and novel algorithm helped predict Mtb peptides that bind HLA-E with improved efficiency [66]. These algorithm-predicted Mtb peptides were able to induce HLA-E-restricted CD8+ T cell proliferation in TB-exposed patients [66].

Crystal structures of HLA-E complexed with the Mtb-derived peptide Mtb44 showed that the HLA-E binding pocket can tolerate peptides with alternative conformations and accommodate both polar and hydrophobic residues [67]. Further study of these findings revealed that although Mtb peptides had lower affinity for HLA-E, they elicited strong T cell responses [68]. These findings suggest that HLA-E can adopt alternate peptide binding positions, bind to a diverse repertoire of peptides, and its binding affinity does not necessarily correlate to the strength of T cell responses. This disconnect between HLA-E peptide affinity and T cell responses raises the possibility of an unknown protein or molecule playing a role in HLA-E peptide loading or an unknown mechanism of peptide exchange.

## 7. HLA-E Antigen Presentation of *Mycobacteria*

Early studies on HLA-E determined that binding the MHC-Ia leader sequence to HLA-E was TAP-dependent [69]. HLA-E also interacted with ER chaperone calreticulin and required tapasin. However, unlike MHC-Ia, HLA-E resides in various cellular compartments during homeostasis, infection, or differentiation and has multiple potential antigen presentation pathways [55]. Camilli et al. showed that the differentiation of monocytes into macrophages induced the expression of HLA-E, but there was only a modest increase in HLA-E molecules at the cell surface [70]. Most of the newly synthesized HLA-E molecules actually colocalized with autophagy–lysosomal vesicles (LC3+ and LAMP1+), indicating a potential compartment for antigens to bind to HLA-E [70]. One potential advantage of HLA-E sampling different cellular compartments is in the setting of infections where antigens are not abundant.

In a study of the HLA-E-dependent presentation of Mtb, Grotzke et al. presented several important findings [71]. First, for Mtb-infected dendritic cells, the presentation of Mtb antigen to an HLA-E-restricted T cell clone required TAP and proteasomal processing. Presentation was only partially blocked by brefeldin A (BFA), which inhibits ER to Golgi transport [71]. This contrasts with MHC-Ia, where BFA caused a substantial reduction in antigen presentation. Second, retrotranslocation, which mainly functions to translocate misfolded proteins in the ER to cytosol, was required for both HLA-E and MHC-Ia antigen presentation, but newly synthesized HLA-E in the ER was not required [71]. Inhibition of retrotranslocation was performed with exotoxin A from *Pseudomonas aeruginosa*, and newly synthesized HLA-E was inhibited by the protein synthesis inhibitor cycloheximide [71]. Strikingly, with cycloheximide, antigen presentation by MHC-Ia was substantially inhibited, while HLA-E presentation remained intact [71]. Finally, purified Mtb phagosomes had more HLA-E than MHC-Ia by Western blot [71]. These phagosomes were capable of activating an HLA-E-restricted T cell clone, confirming that antigen-loaded HLA-E was present [71].

## 8. Clinical Implications of HLA-E and *Mycobacterial* Infections

Vaccination strategies that induce HLA-E-restricted T cells are a feasible approach. Using a rhesus CMV vaccine against Mtb, HLA-E-restricted T cells were elicited in a nonhuman primate model [72]. Although these T cells were not essential for protection against Mtb, the data highlight the potential for HLA-E-targeted vaccination [72]. The importance of HLA-E in protection against mycobacterial infection has been highlighted in multiple other systems. Bian et al. showed that mice deficient in *Qa-1^b^*, a homolog of HLA-E, had increased systemic Mtb burden and a high mortality rate [73]. T cells in *Qa-1^b^* deficient mice had dysregulated production of pro-inflammatory cytokines and decreased Mtb killing [73]. Van Meijgaarden et al. also demonstrated the antimicrobial function of HLA-E-restricted human T cells in inhibiting intracellular Mtb and BCG growth, indicating potential to utilize HLA-E-restricted T cells in therapeutic development [58].

An additional advantage of HLA-E is that it can circumvent any HIV-mediated downregulation of MHC-Ia [74]. La Manna et al. showed that monocyte-derived macrophages from patients with Mtb and HIV coinfection had decreased surface expression of HLA-A2 but stable surface expression of HLA-E [74]. Compared with HLA-A2-restricted clones, HLA-E-restricted clones had increased cytotoxicity and antimicrobial function against coinfected cells. These T cells had increased expression of PD-1, and treatment with antihuman PD-1 monoclonal antibodies promoted expansion and decreased the apoptosis rate of Mtb-specific HLA-E-restricted CD8+ T cells [74]. Given the prevalence of HIV and Mtb coinfection in the developing world, an HLA-E approach to Mtb vaccination could be of great benefit.

## 9. Conclusions

**Figure 1:**
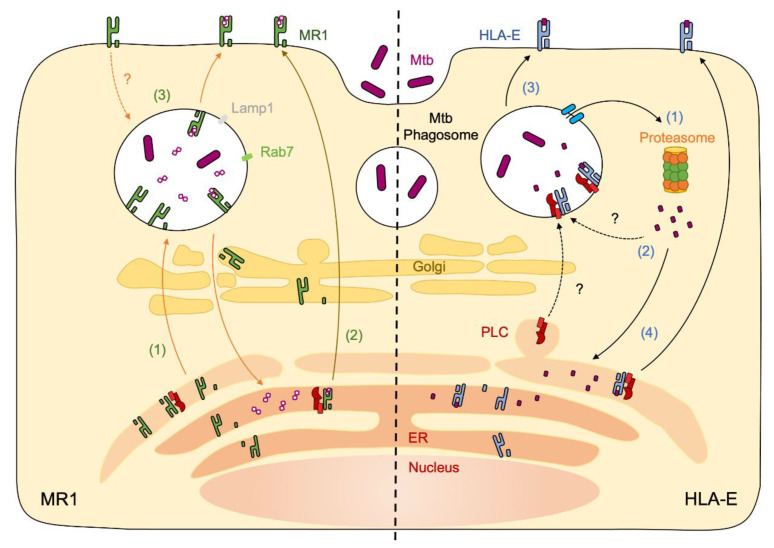
Potential pathways of MR1 and HLA-E antigen presentation of Mtb. MR1: At steady state, newly synthesized MR1 is in the ER along with the PLC. To sample Mtb antigens, MR1 can either traffic to the phagosome (1) or capture mycobacterial antigens in the ER (2). Additionally, it is unknown whether MR1 can recycle from the cell surface to the phagosome (3). HLA-E: Antigens from the Mtb phagosome undergo retrotranslocation to be processed by proteasome in the cytoplasm (1). Mtb-derived peptides then travel back either to the phagosome or to the ER (2). HLA-E interacts with the PLC and is loaded at the phagosome (3) or it is loaded in the ER (4) and then translocates to the cell surface.

Our understanding of nonclassical antigen-presenting molecules has increased rapidly over the last several years. Currently, there are four different nonclassical antigen-presenting molecules with the ability to present Mtb antigens. In this review, we have highlighted MR1 and HLA-E antigen presentation of Mtb antigens. These two antigen-presenting molecules present distinct Mtb antigens, and while we have learned much, the mechanisms by which MR1 and HLA-E capture their Mtb ligands are not fully clear. Unlike HLA-E, which presents peptides and glycopeptides, MR1 presents small microbial metabolites. At present, we do not know whether MR1 antigens are actively shuttled to an MR1-containing compartment or if there is a passive diffusion of antigens. Data from both systems indicate that there are multiple potential pathways for Mtb antigen presentation (Figure 1). These pathways may be an important way for MR1 and HLA-E to sample antigens from different intracellular compartments. Indeed, even MHC-Ia cross presentation has multiple potential pathways [35]. Both the ER–Golgi intermediate compartment and endocytic recycling compartment pathways play a role in transporting MHC-Ia molecules to the cell surface and assist in the loading of antigen from various intracellular compartments, including phagosomes [35]. These pathways could be relevant for MR1 and HLA-E. For example, Ussher et al. showed that the inhibition of endosomal acidification disrupted MR1-depedent antigen presentation in the context of *Escherichia coli* infection, suggesting a role for acidic compartments [75]. In short, the cross-presentation pathways defined in MHC-Ia are an appealing area for further research for understanding MR1 and HLA-E trafficking.

Several areas of MR1 and HLA-E antigen presentation require more study. First, while both MR1 and HLA-E can be loaded with antigen outside of the ER, it is not clear whether there is an endogenous ligand already present. If there is no ligand, what other proteins are involved in stabilizing the antigen-presenting molecule? Another area of inquiry is what governs the translocation of a loaded antigen-presenting molecule to the cell surface. Undoubtedly, there must be mechanisms that facilitate the transport of loaded MR1 and HLA-E to the cell surface, but those mechanisms remain poorly understood. Furthermore, while Mtb employs a broad array of immunoevasion strategies [76], we do not know whether there are any selective mechanisms that perturb MR1 or HLA-E antigen presentation. Finally, and perhaps of greatest importance, there is the question of whether MR1- or HLA-E-specific vaccination strategies can be developed for human infectious diseases. This is an area that warrants further investigation, and a more complete understanding of the antigen presentation pathways will guide therapeutic development.
